# MicroRNA-21: A Critical Pathogenic Factor of Diabetic Nephropathy

**DOI:** 10.3389/fendo.2022.895010

**Published:** 2022-07-05

**Authors:** Shuijiao Liu, Weizhou Wu, Jian Liao, Fuqin Tang, Ge Gao, Jing Peng, Xiujing Fu, Yuqin Zhan, Zhihui Chen, Weifang Xu, Shankun Zhao

**Affiliations:** ^1^ Department of Endocrinology & Department of Gastroenterology, Taizhou Central Hospital (Taizhou University Hospital), Taizhou, China; ^2^ Department of Urology, Maoming People’s Hospital, Maoming, China; ^3^ Department of Nephrology, Jiaxing Hospital of Traditional Chinese Medicine, Jiaxing, China; ^4^ Nursing Department, Taizhou Central Hospital (Taizhou University Hospital), Taizhou, China; ^5^ Department of Clinical Medical School, Taizhou University, Taizhou, China; ^6^ Department of Orthopedics, Taizhou Central Hospital (Taizhou University Hospital), Taizhou, China; ^7^ Department of Urology, Taizhou Central Hospital (Taizhou University Hospital), Taizhou, China

**Keywords:** MicroRNA-21, diabetic nephropahy (DN), target, mechanism, biomarker

## Abstract

Diabetic nephropathy (DN), one of the most common and intractable microvascular complications of diabetes, is the main cause of terminal renal disease globally. MicroRNA-21 (miR-21) is a kind of miRNA early identified in human circulation and tissues. Mounting studies have demonstrated that miR-21 plays an important role in the development and progression of DN. This collaborative review aimed to present a first attempt to capture the current evidence on the relationship between miR-21 and DN. After a systematic search, 29 relevant studies were included for comprehensively and thoroughly reviewing. All these eligible studies reported that miR-21 was up-regulated in DN, whether in serum or renal tissues of human or animal models. MiR-21 exhibited its pathogenic roles in DN by forming a complex network with targeted genes (e.g. MMP-9, Smad7, TIMP3, Cdk6, FOXO1, IMP3, and MMP2) and the signaling cascades (e.g. Akt/TORC1 signaling axis, TGF-β/NF-κB signaling pathways, TGF-β/SMAD pathway, CADM1/STAT3 signaling, and AGE-RAGE regulatory cascade), which resulted in epithelial-to-mesenchymal transition, extracellular matrix deposition, cytoskeletal remodeling, inflammation, and fibrosis. This review highlights that miR-21 is a pivotal pathogenic factor in the development of DN. It may serve as an attractive potential diagnostic, prognostic, and predictive biomarker for DN in clinical practice after further confirmation of the clinicopathological features and molecular mechanisms of miR-21-mediated DN.

## Introduction

Diabetic nephropathy (DN) is a common kidney-related complication of type 1 (T1DM) and type 2 diabetes mellitus (T2DM), affecting 9% of the global adult population (1). According to a recent meta-analysis that included thirty studies, the overall pooled prevalence of DN in China was up to 21.8% [95% confidence interval (CI): 18.5-25.4%] (2). DN occurs in more than 40% of people with diabetes and may develop terminal renal disease (TRD) (3). There are few effective treatments and renal replacement therapy is frequently required. The 5-year survival rate of patients with diabetic terminal kidney disease is only 20% (4). DN-associated heart failure with unchanged ejection fraction has become a major cause of death and morbidity (5, 6). Current treatments for DN include the use of drugs to reduce its progression or renal replacement therapy, neither of which is an effective therapy for DN (7). Therefore, it is imperative to develop an effective, rapid, and non-invasive method to diagnose DN early and predict prognosis to minimize patient mortality and morbidity. In addition, the identification of early biomarkers will help to discover new mechanisms of pathophysiological changes in diabetic kidney damage.

MicroRNAs (miRNAs) are a category of short, endogenous, non-coding RNA molecules, including 19-24 nucleotides. MiRNAs bind the 3′ untranslated region (UTR) of the target gene mRNA to elicit their biological functions, thus promoting the degradation of the mRNA or causing translational repression, eventually implementing the post-transcriptional regulation of gene expression (8). Multiple experimental studies have proved that miRNAs are included in the regulation of many cellular biological processes, such as proliferation, differentiation, and apoptosis. Therefore, the dysregulation of miRNAs is believed to be involved in various pathologies, including the occurrence and progression of DN. It has been reported that most miRNAs exhibit abnormal expression in DN, such as miR-126, and miR-192 (9). In these miRNAs, microRNA-21 (miR-21) is one of the most studied miRNAs whose expression is significantly changed in DN. There has been a lot of research evidence for the role of miR-21 in DN development. Results of a clinical study previously developed by Dey et al. (10) identified a previously undiscovered function of miR-21 for the reciprocal regulation of PTEN (phosphatase and tensin homolog deletions in chromosome 10) levels and Akt/TORC1 (TOR complex 1) activity, which mediated the pathological features of borderline DN. Consistent with this finding, a recent study also demonstrated abnormally elevated levels of miR-21 in DN plasma, with miRNA-21 at 0.01 level in recognizing DN compared to urinary albumin/creatinine ratio (ACR) at 45 mg/gm level. The sensitivity (94.1%) and specificity (100%) were higher (sensitivity 88.2%, specificity 89%), which further suggested that plasma microRNA-21 can be used as an early indicator for the diagnosis and identification of DN (11).

In recent years, the key role of miR-21 in DN has received increasing attention from researchers. In the present study, we present the first attempt to summarize all evidence for the role of miR-21 in DN development in a comprehensive review. Based on the current knowledge, this may help researchers realize the outstanding prognostic and predictive role of miR-21 in DN.

## Overview of MIR-21 in a Variety of Diseases

miRNAs are a category of small regulatory RNAs that quiesce messenger RNAs by binding to their 3’-UTRs. MiRNAs regulate various physiological and pathological processes, including cell differentiation, proliferation, apoptosis, and metabolism, by inhibiting the expression of target genes, inducing mRNA degradation, or inhibiting protein translation (12). As one of the most widely studied microRNAs, microRNA-21 (miR-21) is profoundly expressed in multiple mammalian cell types. It regulates various biological functions like proliferation, differentiation, migration, and apoptosis (13). MiR-21 is omnipresently expressed in normal tissues and cells (14). Previous research has predominantly focused on the relationship between miR-21 and tumors, as miR-21 has been consistently classified to be overexpressed in multiple tumor samples (15), e.g., breast, colon, lung, pancreas, prostate, liver, and stomach cancers (13).

MiR-21 lies on chromosome 17 of Homo sapiens and is extremely conserved. Its promoter stated by Fujita et al. has some conserved enhancer elements containing the binding sites for multiple targeted genes, e.g. STAT3, p53, AP-1, Ets/PU1, C/EBPa, NFI, and SRF (16, 17). At the cellular level, miR-21 is found to be located in the cytoplasm (18) as well as in the exosomes (19). At the organ level, miR-21 can be detected in various organs and tissues, e.g. bone marrow, peripheral blood, kidney, intestine, liver, colon, lung, and thyroid (20). Functionally, miR-21 is involved in post-transcriptional gene silencing by binding to the 3’ untranslated region (UTR), thereby regulating its targets. Using computational algorithms, it was found that 175 genes involved in biological regulation, cellular and metabolic processes were all regulated by miR-21 (21), while, relatively few genes were experimentally validated (13).

Currently, there are several review articles that have illustrated the association between miR-21 expression and human malignancies [e.g. melanoma (22), osteosarcoma (23), cervical cancer (24), and pancreatic cancer (25)], central nervous system disorders (26), arthritis (27), and skin diseases (28). Whereas, there is currently no relevant review article addressing the clinical implications and the underlying molecular mechanisms of miR-21 in DN, so a comprehensive review of all the current evidence on this issue is warranted.

## Literature Search and the Characteristic of the Included Studies

**Figure 1:**
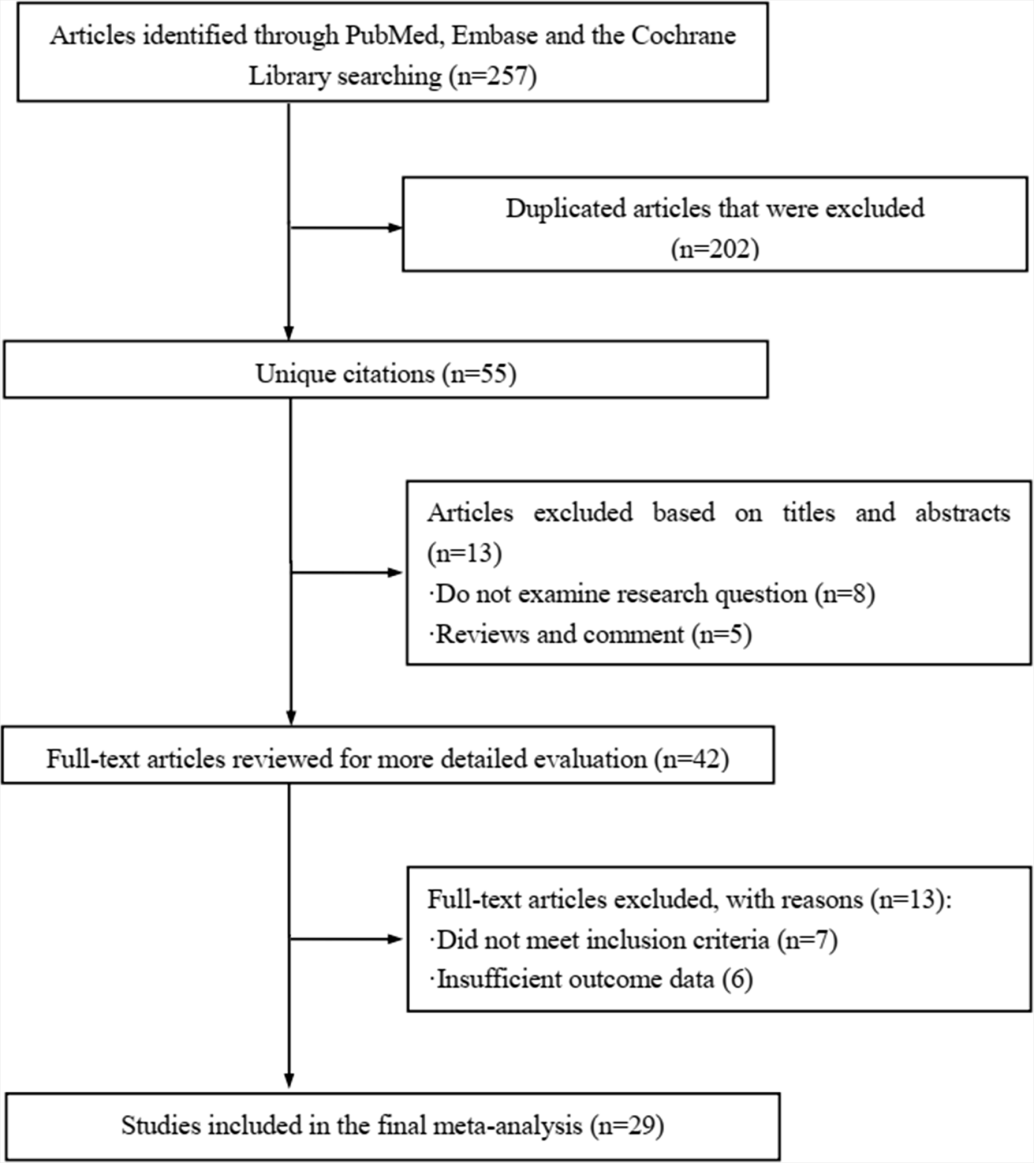
Flow chart of study selection.

**Figure 2:**
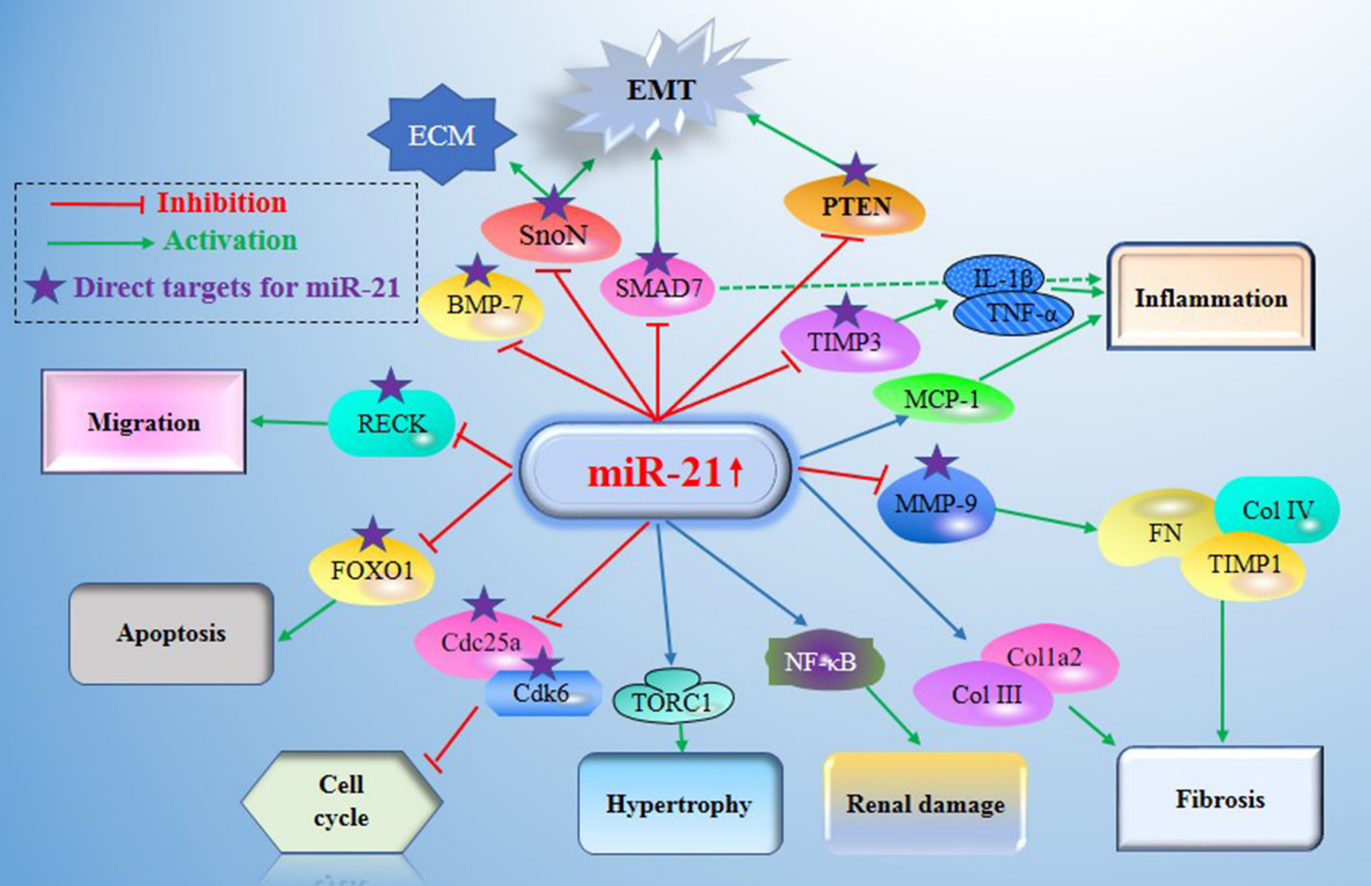
Main mechanisms of miR21 in diabetic nephropathy (DN). miR-21 exerts its central roles in DN by constituting a complex network with the direct target genes (e.g. MMP-9, Smad7, TIMP3, Cdk6, FOXO1, IMP3, and MMP2) and the signaling cascades. DN, Diabetic nephropathy; miR-21, MicroRNA-21; TORC1, TOR complex 1; PTEN, phosphatase and tensin homolog deleted in chromosome 10; TGF-β1, Transforming growth factor-β1; TIMPs, tissue inhibitors of metalloproteinases; MMPs, matrix metalloproteinases; BMP-7, Bone morphogenetic protein 7; TIMP3, tissue inhibitors of metalloproteinase 3; MCP-1, monocyte chemoattractant protein 1; SnoN, Skirelated novel protein; EMT, epithelial-to-mesenchymal transition; ECM, extracellular matrix; RECK, reversion Inducing cysteine rich protein with Kazal Motifs; FOXO1, forkhead box O1; Cdc25a, Cell division cycle 25a; Cdk6, cyclindependent kinase 6; NF-Kb, nuclear factor κB.

The literature review was commencing on the six common-used databases, e.g. MEDLINE, EMBASE, Google Scholar, Cochrane Library, Web of Science, and PsychINFO, to explore the related studies reporting the relationship between miR-21 in DN. The searching strategy in the MEDLINE by using the keywords was: ((((((miR-21) OR (microRNA-21)) OR (hsa-mir-21)) OR (miR-21-5p)) OR (miR-21-3p)) OR (miR21a)) AND ((((((((((((((((((“Diabetic Nephropathies”[Mesh]) OR (Nephropathies, Diabetic)) OR (Nephropathy, Diabetic)) OR (Diabetic Nephropathy)) OR (Diabetic Kidney Disease)) OR (Diabetic Kidney Diseases)) OR (Kidney Disease, Diabetic)) OR (Kidney Diseases, Diabetic)) OR (Diabetic Glomerulosclerosis)) OR (Glomerulosclerosis, Diabetic)) OR (Intracapillary Glomerulosclerosis)) OR (Nodular Glomerulosclerosis)) OR (Glomerulosclerosis, Nodular)) OR (Kimmelstiel-Wilson Syndrome)) OR (Kimmelstiel Wilson Syndrome)) OR (Syndrome, Kimmelstiel-Wilson)) OR (Kimmelstiel-Wilson Disease)) OR (Kimmelstiel Wilson Disease)). In addition, the reference list was reviewed to identify more relevant studies. Routine data collection forms were used to extract relevant data from the included studies, such as first author name and citations, year of publication, study/subject, roles of miR-21, mechanisms involved, target genes, related signaling pathways, and each primary results from one eligible study. Twenty-nine studies published in 2011-2021 were included (4, 10, 11, 29–54). **Figure 1** showed the flowchart of the studies reviewed. The types of the involved studies were either clinical trials or experimental studies (e.g. *in vitro* or *in vivo*). The research contents among the clinical studies included kidney tissues, plasma, and urine of the DN patients. All of the eligible studies showed elevated levels of that miR-21 expression in DN patients. The underlying mechanisms reported in the eligible studies mainly include proliferation, migration, invasion, apoptosis, cell cycle, epithelial-to-mesenchymal transition (EMT), cytoskeletal remodeling, oxidative stress, and M2 macrophage dysfunction. The direct targets of miR-21 include Matrix metalloproteinases (MMP-9), inhibitory Smad (Smad7), inhibitors of metalloproteinases (TIMP3), cyclin-dependent Kinase (Cdk6), and forkhead box O1 (FOXO1). The relevant signaling cascades included Akt/TORC1 signaling axis, TGF-β, and NF-κB signaling pathway, TGF-β/SMAD pathway, CADM1/STAT3 signaling, KEGG pathway, and AGE-RAGE regulatory cascade. **Table 1** listed the characteristics of the eligible studies reporting miR-21 in DN. **Figure 2** displayed the main mechanisms of miR21 in DN.

**Table 1:** Summary of miR-21 in diabetic nephropathy (DN) reported in the relevant studies.

Study/Reference	Research objects	MiR-21 expression	Involved Mechanism	Target Gene	Associated pathways	Main findings
*Clinical trials reporting the roles of miR-21 in DN*						
(31)	Patients plasma and urine	Up	Clinical study	Clinical study	Clinical study	In type 1 diabetic patients’ plasma and urine, miRNA levels of miR-21 and miR-210 were significantly increased. They discovered an AUC of 0.78 for urine miR-21 using an adjusted ROC-curve analysis.
(36)	Plasma	Up	Clinical study	Clinical study	TGB-β1↑	miR-21-5p were linked to a more than 2.5-fold increase in the risk of ESRD (P ≤ 0.005).
(38)	Patients Serum	Up	Clinical study	Clinical study	Clinical study	Only miR-21 levels in low-eGFR patients were considerably higher than in high-eGFR patients. MiR-21 could be biomarkers for DN progression and represent DN pathogenesis.
(45)	Patients plasma	Up	Clinical study	Clinical study	AGE-RAGE, TGF-β1, and relaxin signaling pathways↑	In DKD cases, miR-21-3p and miR-378a-5p expression were negatively connected with eGFR (r=0.633, P = 0.004; and r=0.455, P = 0.044, respectively). The expression of MiR-21-3p was likewise linked to serum creatinine levels (r = 0.616, P = 0.004).
(48)	Patient Serum and urine	Up	Clinical study	Clinical study	Clinical study	Urinary miR-21-5p, let-7e-5p, and miR-23b-3p were significantly elevated in T2DKD compared to T2DNRF in the discovery cohort. Independent validation showed up-regulation of miR-21-5p in T2DKD (2.13-fold, p = 0.006) and CCKD (1.73-fold, p = 0.024) replication cohorts.
(11)	Patients plasma	Up	Clinical study	Clinical study	Clinical study	MiR-21 in the blood can be used as an early indicator of diabetic nephropathy in people with T1DM. For detecting DN, plasma miRNA-21 at a concentration of 0.01 demonstrated a higher sensitivity (94.1%) and specificity (100%) than ACR at 45 mg/gm. 88.2% sensitivity and 89% specificity.
(53)	Patients serum and urine	Up	Clinical study and experimental study	MMP9	KEGG↑	MMP9 and PTEN were discovered to be targets of hsa-miR-21-5p in silico study, implying that metformin modulates MMP9 expression in T2DM and DN patients *via* hsa-miR-21-5p. Metformin regulates MMP9 *via* miR-21, which is involved in the progression and pathogenesis of diabetic immune complications by preventing thrombus formation, phagocytosis involvement, and cellular matrix and immune complex degradation.
*Direct target of miR-21: PTEN*						
(10)	Mouse tissues	Up	Phosphorylation Transcription	PTEN	Akt/TORC1 ↑	Up-regulation of miR-21 promoted renal fibrosis. High glucose-sensitive miR-21 expression increased Akt phosphorylation, resulting in PRAS40 inactivation and therefore enhancing TORC1 activity.
(35)	Patients tissues	Up	Proliferation	Smad7 and PTEN	TGF-β/smad3/PI3K↑	MiR-21 levels influence Smad7 and PTEN protein translation, and miR-21 upregulation is linked to the degree of fibrosis and the rate of decrease in renal function in human DN.
*Direct target of miR-21: MMP-9 and MMP-2*						
(4)	Mouse tissues	Up	Downregulated MMP-9; increased TIMP-1expression;	MMP-9	NA	MiR-21 expression was shown to be positively connected with ACR and the glomerular fibrosis index (GSI), but negatively correlated with Ccr. MiR-21 has a role in the DN process, which promotes urinary protein excretion and exacerbates renal function impairment. By modulating the production of TIMP-1 and MMP-9 in DN, miR-21 promotes ECM deposition.
(40)	Mouse tissues and serum/urine	Up	Migration	MMP-9	NA	MiR-21 was found to be the most important miRNA in fibrotic diseases, with its expression being up-regulated in nephropathy. By down-regulating miR-21, the Hyp promotes MMP-9 protein production.
(42)	Mouse tissues	Up	Proliferation invasion	MMP-9	CADM1/STAT3	The levels of miR-21 in cardiac tissue were found to be positively correlated with ACR (r = -0.870, P = 0.003), suggesting that miR-21 may play a role in the pathogenesis of cardiac fibrosis with DN by targeting MMP-9.
(52)	Patients; cells; mouse	Up	Oxidative stress, RECK suppression, EMT and migration	MMP2	TRAF3IP2↑	In cultured kidney proximal tubule cells, the SGLT2 inhibitor EMPA reduces HG-induced oxidative stress, RECK suppression, and EMT, and these beneficial effects are linked to lower expression of several proinflammatory and profibrotic mediators, including TRAF3IP2, NF-B, p38MAPK, and miR-21.
*Direct target of miR-21: Smad7*						
(29)	Mouse tissues	Up	Feed-forward loop phosphorylated	Smad7	TGF-β and NF-κB signalling pathways↑	Upregulation of miR-21 lowers Smad7 abundance, high glucose-induced fibrosis, and NF-B-mediated inflammation, making it a key component of high-glucose-induced *signaling*.
(32)	Mouse tissues	Up	Proliferation	Smad7	TGF-β/SMAD↑	SMAD7 is regulated by miR 21, and the protein level of SMAD7 in rat renal tubular epithelial cells correlates negatively with miR 21 expression.
(33)	Mouse and patients tissue	Up	Proliferation,EMT	Smad7	TGF-β1↑	Targeting miR-21 may be a better approach to directly limit TGF-β1-mediated fibrosis in DN, as it can contribute to TGF-β1-induced EMT by blocking target smad7.
(33)	Mouse tissues	Up	Proliferation, migration, transcription and EMT	Smad7	TGF-β1/smad3↑	By suppressing target smad7, miR-21 and TGF-β1/smad3 produced a double-positive feedback loop to accelerate renal tubular EMT.
(37)	Mouse tissues	Up	p-Smad3	Smad7	NA	C66 can partially alleviate diabetes-induced kidney fibrosis by reversing elevated p-Smad3 levels *via* downregulating miR-21.
(39)	Mouse tissues and serum	Up	Proliferation	Smad7	TGF-β/Smads and NF-κB↑	The alterations in tissue miR-21 may be reflected in serum miR-21. With the progression of DN, serum and renal tissue miR-21 levels were dramatically raised. Serum miR-21 was found to be closely linked to renal shape and function, suggesting that serum miR-21 could be used as a possible DN diagnostic biomarker.
(42)	Mouse tissues and serum/urine	Up	Proliferation and EMT	Smad7	β-catenin pathway/ (TGF)-β1/Smads pathway ↑	Treatment with AS-IV reduced the effects of overexpressed miR-21, which enhanced podocyte dedifferentiation and MC activation. Furthermore, in the process of podocyte dedifferentiation and MC activation, overexpression of miR-21 activated the catenin pathway and the TGF-β1/Smads pathway, which was blocked by AS-IV therapy.
(49)	Mouse Cells and serum	Up	Transcriptional activation	Smad7	TGF-β1/Smad3↑	MiR-21 modulates p-Smad3 (Ser423/425), EMT, and ECM deposition *via* Smad7 regulation under high glucose and diabetes circumstances. *In vitro* and *in vivo*, BMP-7 is implicated in the antifibrotic process in DN through modulating miR-21/Smad7 and influencing TGF-β1/Smad3 signaling.
(46)	Mouse tissues and serum/urine	Up	Transcriptional	Smad7	TGF‐β1/Smad3↑	Overexpression and inhibition of miR-21 enhanced and reduced EMT and ECM deposition, respectively, without changing SnoN levels, according to our findings. SnoN, through downregulating miR-21, reduces the development of DN as well as renal fibrosis.
*Direct target of miR-21: TIMP3*						
(30)	Mouse and patients tissue	Up	Timp3 messenger RNA destruction	TIMP3	Timp3 messenger RNA destruction↑	Only miR-21 expression was higher in diabetic patients’ kidney biopsies compared to healthy controls in human samples. In diabetic kidneys, the expression of specific TIMP3-targeting miRs is higher than in healthy controls.
(44)	Mouse and patients tissues/blood and urine	Up	Apoptosis, Proliferation, migration	TIMP3	NA	In DN patients’ serum and renal tissues, miR-21 expression was upregulated. Increased miR-21 relieved TIMP3’s inhibitory effects on inflammatory responses and podocyte death. STZ-induced renal damage in DN rats was reduced when miR-21 was inhibited.
(46)	Mouse tissues	Up	NA	IMP3	RNA silencing pathway↑	Overexpression of the lncRNA TUG1 inhibits cell fibrosis in high glucose-stimulated NRK-52E cells and renal fibrosis in DN mice by targeting the miR-21 and promoting the expression of TIMP3.
*Other direct target of miR-21*						
(41)	Patients and Mouse tissues/serum/urine	Up	Proliferation migration	Cdk6	AP-1	New miR-21 targets in mesangial cells include cell division cycle 25a (Cdc25a) and cyclindependent kinase 6 (Cdk6). Cdc25a and Cdk6 were repressed by miR-21, resulting in slowed cell cycle progression and consequent mesangial cell hypertrophy. By modulating phosphatase and tensin homolog, miR-21 improved podocyte motility (Pten). MiR-21 regulates mesangial cell hypertrophy and podocyte phenotype.
(50)	Mouse serum and urine	Up	Apoptosis podocytes	FOXO1	NA	In DN mice, we discovered that Atr reduced kidney damage by reducing miR-21 expression and promoted autophagy. In high hyperglycemia -treated podocytes, deletion of miR-21 decreased apoptosis and increased autophagy.
(47)	EDN samples	Up	NA	NA	TGFβ1↑	TGFβ1 could play a role in DN advancement *via* regulating miR-21-5p, miR-146a-5p, and RAD21.
(54)	Mouse; cell; blood	Up	Restraint reduced ROS	NA	NA	A20 in DN can regulate pyroptosis-mediated podocyte damage by MiR-21-5p in macrophage-derived EVs. Through targeted suppression of A20, miR-21-5p in macrophage-derived EVs can raise the inflammasome NLRP3, caspases-1, and IL-1β associated to pyroptosis, as well as increase the generation of ROS, causing podocyte damage.

DN, Diabetic nephropathy; T2D, type 2 diabetes; miR-21, MicroRNA-2; TORC1, TOR complex 1; CCKD, chronic kidney disease; PTEN, phosphatase and tensin homolog deleted in chromosome 10; TGF-β1, Transforming growth factor-β1; TIMPs, tissue inhibitors of metalloproteinases; MMPs, matrix metalloproteinases; BMP-7, Bone morphogenetic protein 7; GSI, glomerular fibrosis index; eGFR, estimated glomerular filtration rate; Ccr, creatine clearance ratio; AGE, advanced glycation end products; MC, mesangial cell; AS-IV, astragaloside IV; TIMP3, tissue inhibitors of metalloproteinase 3; SnoN, Skirelated novel protein; FN, fibronectin; EMT, epithelial-to-mesenchymal transition; ECM, extracellular matrix; RECK, reversion Inducing cysteine rich protein with Kazal Motifs; EMPA, empagliflozin; FOXO1, forkhead box O1; Cdc25a, Cell division cycle 25a; Cdk6, cyclindependent kinase 6; Atr, Atrasentan; SGLT, sodium-glucose co-transporters; NF-Kb, nuclear factor κB; SGLT, sodium-glucose co-transporters; ROS, reactive oxygen species; NLRP3, nucleotide-binding domain, leucine-rich-containing family, pyrin domain-containing-3. ↑: up-regulation; NA, not available.

## Clinical Significances of miR-21 in DN

Fourteen included studies that stated detailed clinical information on miR-21 expression levels in DN. All qualified clinical studies indicated that miR-21 was upregulated in DN patients.

### miR-21 Expression Was Higher in Renal Biopsy of Patients With DN, Associated With Renal Fibrosis

The histopathological changes of miR-21 expression in DN renal biopsy are significant, which can directly reflect the key role of miR-21 in the occurrence and development of DN. All the fourteen included studies provided information on the expression of miR-21 in pathological sections and consistently showed that miR-21 was up-regulated in DN renal tissues. However, the correlation between miR-21 and clinical features of DN was slightly different among different studies. Wang et al. (4) reported that the expression of miR-21 was remarkably elevated in kkay mice compared with the control C57BL mice. Besides, the level of miR-21 was positively associated with the level of TIMP1, Col IV, and fibronectin, but negatively associated with the expression of MMP-9. Moreover, the miR-21 expression also correlated with the creatine clearance (Ccr) and the urine albumin creatine ratio (ACR). In McClelland’s study (35), they collected kidney biopsies from 35 patients with varying degrees of T2DM-related kidney injury and 8 control subjects. Renal biopsies showed that miR-21 expression was significantly increased in patients with fast progression of renal function decline (median time to dialysis 18 months) compared with those diabetic patients with slower progression (time to dialysis 60 months). Based on the histological analysis of the kidneys and the pathology of the degree of renal interstitial sclerosis, the degree of tissue, fibrosis, and moderate to severe fibrosis within the patient’s tissue will be further determined. Meanwhile, miR-21 levels were elevated with increased renal fibrosis. MiR-21 levels were highest in patients with moderate to severe fibrosis. Wang et al. (51) stated that overexpression and inhibition of miR-21 respectively promoted and inhibited EMT and extracellular matrix (ECM) deposition, respectively, without affecting the level of skirelated novel protein (SnoN). The result showed that SnoN inhibited the development of DN as well as renal fibrosis by downregulating miR-21.

### The Plasma and Urine miR-21 Was Significantly Increased in Patients With DN, Which Was Associated With the Clinical Features of DN

It has been reported that increased miR-21 expression was observed not only in DN tissues but also in the plasma and urine of DN patients. Lots of studies have shown that miRNAs have the probability to be exposed as effective biomarkers in body fluids. It was suggested that miR-21 was positively correlated with ACR and glomerular fibrosis index (GSI), but was negatively associated with Ccr (4). MiR-21 involves in the process of DN, promotes urinary protein egestion, and aggravates renal function damage. Wang et al. (39) found that miR-21 in serum and kidney tissue was significantly increased with the progression of DN. Serum miR-21 was significantly compliant with tissue miR-21 changes during DN development. What’s more, miR-21 in serum was positively correlated with GBM, GA, ACR, and CCF, and negatively correlated with Ccr. Chen et al. (44) found that the expression of miR-21 was up-regulated in serum and renal tissue of DN patients, and the elevation of miR-21 alleviated the inhibitory effect of TIMP3 on inflammatory response and podocyte apoptosis. Inhibition of miR-21 attenuates renal injury in STZ-induced DN rats. Zang et al. (48) found that urinary miR-21-5p, let-7e-5p, and miR-23b-3p were significantly upregulated in T2DKD patients compared to T2DNRF in the discovery cohort (P < 0.05). Likewise, miRNAs in urine were successfully detected and remained stable over time (55). Osipova et al. (31) suggested that urinary miR-21 emerged as a latent noninvasive biomarker for type 1 diabetes and may identify patients at high risk for future renal and cardiovascular damage. This study examined the concentrations of miR-21, miR-126, and miR-210 in plasma by the quantitative RT-PCR, and pointed out that cell division cycle 25a (Cdc25a) and cyclin-dependent kinase 6 (Cdk6) were the novel targets of miR-21 in mesangial cells. MiR-21-mediated inhibition of Cdc25a and Cdk6 caused the impairment of cell cycle progression. The authors further found that the plasma and urine levels of miR-210 were higher in patients with type 1 diabetes, and the AUC was recorded at 0.78 by adjusting the ROC-curve analysis. In the study of Assmann et al. (45), among the 48 miRNAs screened and analyzed, nine miRNAs (hsa-miR-16-5p, hsa-miR-21-3p, hsa-miR-29a-3p, hsa-miR-141-3p, hsa-miR-192-5p, hsa-miR-204-5p, hsa-miR-215-5p, hsa-miR-378a-5p, and hsa-miR-503-5p) were differentially expressed between diabetic kidney disease cases and T1DM controls. More importantly, the expressions of both miR-21-3p (r = - 0.633, P = 0.004) and miR-378a-5p (r = - 0.455, P = 0.044) were negatively associated to eGFR, while positive association was observed between miR-378a-5p expression and urinary albumin excretion levels (r = 0.338, P = 0.049), as well as the association between miR-21-3p and serum creatinine levels (r = 0.616, P = 0.004). These studies clarify the fact that miR-21 in the plasma and urine of DN patients is higher than that of healthy controls, and miRNAs can be detected as available biomarkers in body fluids.

## Molecular Mechanisms of miR-21 in DN

Since the aforementioned clinical studies have demonstrated a causal relationship between miR-21 level and DN, understanding more about the biological function of miR-21 and its potential mechanisms in DN is profound for the researchers. MiR-21 was up-regulated in most DN and is therefore thought to play a pathogenic role in DN. MiRNAs are tiny non-coding RNA molecules that are contained in post-transcriptional gene regulation and multiple of which have been researched in chronic kidney disease. Extensive pre-clinical and clinical studies have emphasized the underlying role of miRNAs in the pathogenesis of hypertensive nephrosclerosis, diabetic nephrosclerosis, glomerulonephritis, renal tubulointerstitial fibrosis, and some associated cardiovascular complications.

### Inflammatory Microenvironment Underlies the Effect of miR-21 in DN Genesis

Inflammation plays an essential role in the pathogenesis of DN, which involves immune cells and the microenvironment (56). MiR-21-5p knockdown also ameliorated renal inflammation, i.e. infiltration by F4/80 positive macrophages, probably *via* weakening the expression of MCP-1, a well-known chemokine that mainly causes monocytes and macrophages to sites of damage. In addition, miR-21-5p silencing prevented podocyte loss and reduced albuminuria compared with control mice (41). It was also suggested that knockdown of miR-21-5p by using locked nucleic acid-anti-miR-21-5p in STZ-induced murine diabetes, leads to decreased interstitial fibrosis due to the reduction of the expression of the collagen genes Col1a2 and Col III. A previous study developed by Zhong et al. demonstrated that Smad7 was a direct target of miR-21 during renal inflammation (29). Overexpression of miR-21-5p in rat renal tubular epithelial cells and mesenchymal cells reduced the expression of Smad7, while knockdown of miR-21-5p restored the level of Smad7 (29). The authors further found that Smad7 siRNA administration significantly enhanced the level of TNF-α and Il-1β induced by high glucose. Targeting Smad7 might be a mechanism by which miR-21 regulated renal injury in the type 2 diabetes animal model. It was reported that knockdown of Smad7 in-vitro enhanced the expression of inflammatory cytokines, indicating that Smad7 played a protective role in renal inflammation-induced DN (39, 44). Chen et al. (44) showed that the expression of miR-21 was upregulated in serum and kidney tissues of DN patients, kidney tissues of STZ induced DN rats, and HG-treated podocytes. In STZ-induced DN rats, MiR-21 depletion could inhibit the secretion of pro-inflammatory factors (IL-1β, TNF-α) and relieve kidney damage. Increased miR-21 attenuated the inhibitory effect of TIMP3 on inflammatory responses and podocyte apoptosis. In addition to DN, many previous studies reported that miR-21-mediated inflammation was involved in various diseases, such as myocardial injury, stroke, psoriasis, and carcinogenesis (57, 58).

**Figure 3** showed the proinflammatory molecular mechanisms at the kidney level where miR-21 was involved in the development of DN.

**Figure:**
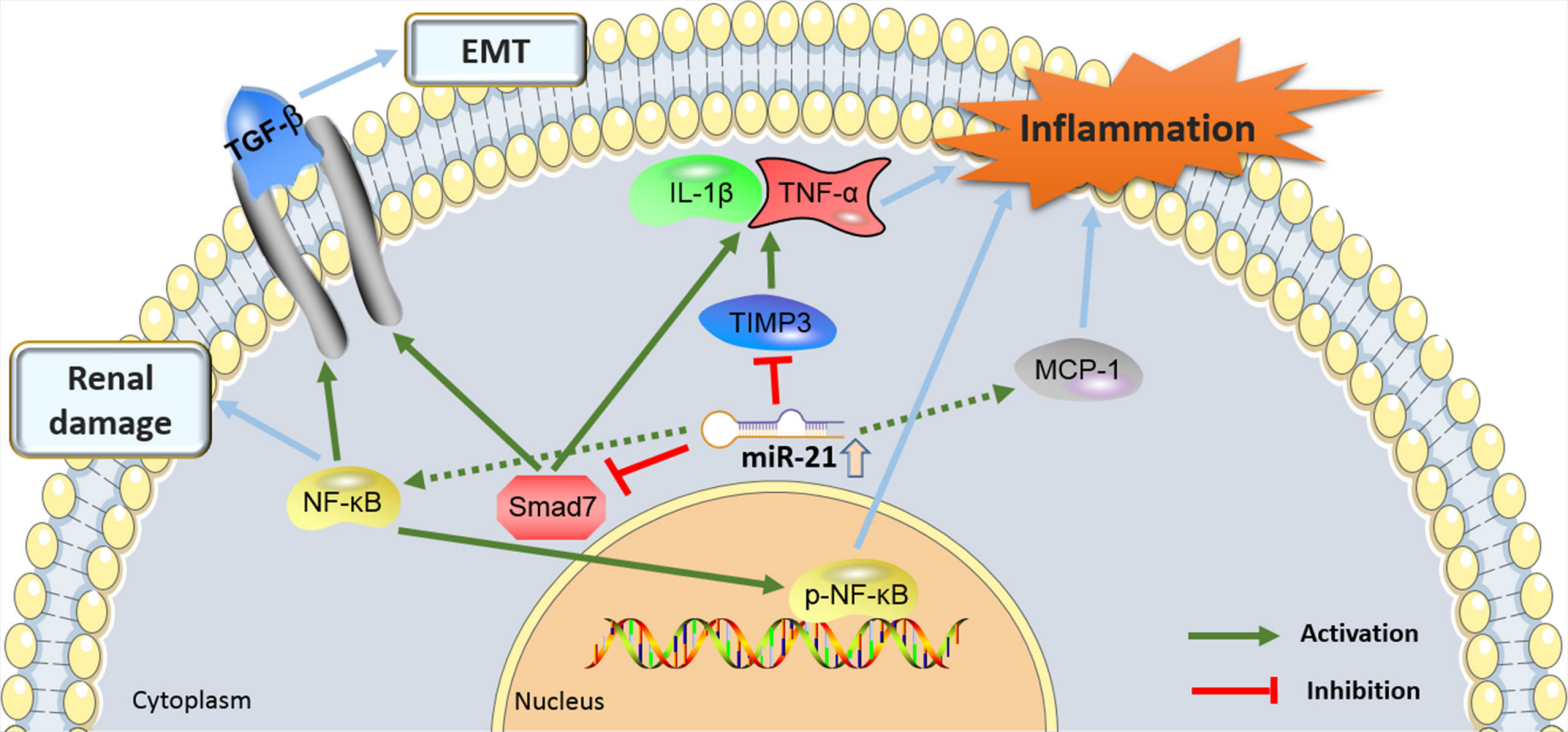
Figure 3. The proinflammatory molecular mechanisms by which the miR-21 and its target genes contributes to the pathogenesis of diabetic nephropathy (DN). miR-21, MicroRNA-21; TGF-β, Transforming growth factor-β; TIMP3, tissue inhibitors of metalloproteinase 3; EMT, epithelial-to-mesenchymal transition; NF-Kb, nuclear factor κB; MCP-1, monocyte chemoattractant protein 1.

### Roles of EMT in miR-21-Regulated DN

EMT plays an important role in DN complicated with renal interstitial fibrosis (RIF). Smad7, an inhibitory Smad, is a downstream signaling pathway of TGF-b1 that inhibits EMT. The physiological function of miR-21 is closely related to EMT and RIF. EMT and ECM deposition in renal tubular epithelial cells contribute greatly to the pathogenesis of DN, but the potential mechanisms remain unclear. Bone morphogenetic protein 7 (BMP-7) restrains EMT and ECM accumulation in kidney tubular epithelial cells cultured with high glucose. MiR-21 not only downregulates Smad7 but also promotes EMT and ECM deposition. Under the conditions of high glucose and diabetes, miR-21 controls p-Smad3 (Ser423/425), EMT, and ECM deposition modulation through Smad7. BMP-7 mRNA and protein levels decreased, miR-21 content increased, Smad7 mRNA increased, and protein expression decreased. *In vitro* and *in vivo*, BMP-7 is involved in the anti-fibrotic process of DN by modulating miR-21/Smad7 and regulating the TGF-β1/Smad3 signaling pathway (47). Three studies (33, 34, 43) revealed that EMT might be one of the vital pathomechanisms potential the miR-21-mediated DN miR-21 overexpression, which could cause TGF-b1-induced EMT by inhibiting the smad7 target, targeting miR-21 might be a better alternative to directly inhibit TGF-b1-mediated fibrosis in DN. The miR-21 inhibitor can reduce fibrosis markers (col-IV, FN) expression and collagen fibers deposition, ameliorating renal structure and function. In Khokhar et al.’s (53) study, they showed MMP9 and PTEN to be the miR-21-5p targets in-silico analysis and suggested that metformin regulates MMP9 expression in T2DM and DN patient populations through hsa-miR-21-5p. This is the first study that showed metformin regulating MMP9 through miR-21, which is involved in the pathogenesis and progression of diabetic immune complications by preventing thrombus formation, the phagocytosis process, and degradation of both cellular matrix and immune complex. This evidence shows that EMT might contribute to the development of DN and the progression of miR-21-mediated DN. Also, it was reported that miR-21-mediated EMT played roles in various diseases, i.e., breast cancer, osteosarcomas, and endometrial fibrosis (59, 60).

### MiR-21 Serves as a Pathogenic Factor by Affecting the Renal Cellular Function

A large number of experimental studies have shown that a variety of miRNAs play an important role in the occurrence and development of DN by promoting the proliferation, migration, and invasion of the cells. Das et al. (52) recently argued suppression of RECK (Reversion Inducing Cysteine Rich Protein with Kazal Motifs), a membrane-anchored endogenous MMP inhibitor and anti-fibrotic mediator, in the kidneys of DB/db mice, a model of diabetic nephropathy disease, as well as in human renal proximal tubule cells (HK–2) treated with high glucose (HG). They further indicated that the SGLT2 inhibitor empagliflozin (EMPA) transposed these effects. Little is known about the mechanisms of RECK inhibition under hyperglycemic conditions and its deliverance by EMPA. HG (25 mM) inhibited RECK expression in HK-2 cells. Additionally, mechanistic studies revealed that HG-induced superoxide and hydrogen peroxide production, oxidative stress-dependent TRAF3IP2 upregulation, activation of NF-kB, and p38 MAPK, inflammatory cytokine expression (IL-1β, IL-6, TNF-α, and MCP-1), miR-21 induction, MMP2 activation, and RECK inhibition. Furthermore, gain-of-function RECK suppressed HG-induced MMP2 activation and HK-2 cell migration. The same as HG, advanced glycation end products (AGE) stimulated TRAF3IP2 and inhibited RECK, which were suppressed by EMPA. Significantly, EMPA treatment enhanced all of these deleterious effects and suppressed EMT and HK-2 cell migration. Taken together, these findings show that hyperglycemia and linked AGE inhibit RECK expression by way of oxidative stress/TRAF3IP2/NF-KB and p38 MAPK/miR-21 induction. Downregulation of miR-21 inhibited apoptosis and induced autophagy in HG-treated podocytes was found by Wang et al. (Wang et al., 2018). To alleviate renal injury, atrasentan (Atr) inhibited the miR-21 expression and promoted autophagy in DN mice. Moreover, FOXO1 was detected as a target of miR-21. MiR-21 exhibited its pro-apoptosis and anti-autophagy effects by targeting FOXO1 in HG cultured podocytes. Atr enhanced FOXO1 expression by downregulating miR-21 in HG-cultured podocytes. Atr attenuated kidney damage in DN mice and mitigated HG-mediated apoptosis increase and autophagy inhibition in podocytes by adjusting the miR-21/FOXO1 axis, further clarifying the molecular basis by which Atr hampered DN progression. As Liu et al. (49) pointed out, under high glucose and diabetes conditions, BMP-7 mRNA and protein levels were decreased, miR-21 amounts were raised, and Smad7 mRNA were was decreased. The expression of Smad7, which induces the expression of Smad7 mRNA under HG conditions is affected by the ubiquitin-proteasome system; in the meantime, upregulation of E3 ubiquitin ligase Smurf1, Smurf2, and Arkadia occurs, elevating the degradation of the Smad7 protein. This is definite because protein degradation is more vigorously than transcriptional activation, causing a decrease in Smad7 protein levels.

### Other Potential Mechanisms Underlay miR-21-Mediated DN

As shown in **Table 1**, in addition to the above-supposed molecular mechanisms, other pathomechanisms might also involve the action of miR-21-mediated DN, including cytoskeletal remodeling, glycolytic metabolism metergasis, cell cycle, oxidative stress damage, anti-apoptosis, M2 macrophages metergasis, and the dysregulation of the affected signaling pathways. Zhong et al. (29) explained the signaling pathways correlated with renal fibrosis and inflammation, for instance, the TGF-β and NF-КB pathways, in diabetic kidneys. TGF-β1 protein was upregulated in kidneys from DB/db mice at 10 weeks of age. Meanwhile, both phosphorylated Smad3 and NF-κB-p65 were raised, suggesting that both TGF-β and NF-κB pathways were alive during diabetic renal damage, TGF-β1 protein, phosphorylated Smad3, and NF-κB-p65 were decreased after the miR-21 KD plasmids treatment, suggesting that miR-21 inhibits the signaling pathways activation of the TGF-βand NF-κB. In light of these findings, renal miR-21 restored Smad7 levels and inhibited the TGF-β and NF-κB signaling pathways activation.

MiR-21-5p expression is elevated in serum or kidneys of both DN patients and DN mice (48). This increased expression in DN correlates with tubulointerstitial fibrosis, renal damage, and decreased eGFR. In-vivo targeting of miR-21-5p using ultrasound-microbubble-mediated gene delivery, locked nucleic acid, or lentiviral vectors has been applied to validate its role in DN development. Furthermore, miR-21-5p also impacts CKD-related cardiovascular events. For instance, in 5/6 nephrectomized rats developed concentric left-ventricular hypertrophy that was not driven by volume expansion within 4 to 5 weeks after CKD induction (61). Kölling et al. (41) suggested that miR-21 acted as a powerful regulator of mesangial cell hypertrophy and podocyte phenotype increasing podocyte motility by adjusting PTEN. MiR-21-mediated repression of Cdc25a and Cdk6 caused impaired cell cycle progression and pursuant mesangial cell hypertrophy. Cdc25a and Cdk6 have been identified as the novel miR-21 targeted genes in mesangial cells. Dey et al. (10) found that miR-21 is the molecular link between high glucose and PTEN inhibition. Renal cortex from OVE26 type 1 diabetic mice displayed significantly elevated levels of miR-21 relevant to reduce PTEN and enhance fibronectin content. Overexpression of miR-21 simulated the action of high glucose, including a reduction in PTEN expression and an accompanying increase in Akt phosphorylation. By comparison, the expression of miR-21 Sponge could restrain endogenous miR-21 and block high glucose-induced PTEN downregulation and Akt phosphorylation. Intriguingly, high glucose-stimulated miR-21 inactivated PRAS40, a negative regulator of TORC1. Eventually, miR-21 raised high glucose-induced TORC1 activity, causing renal cell hypertrophy and fibronectin expression.

## Conclusion and Perspectives

To the best of our knowledge, this comprehensive review is the first study to summarize all the current evidence on the relationship between miR-21 and DN. All the 29 relevant studies demonstrated that miR-21 is a pathogenic factor in DN development. MiR-21 expression is dramatically upregulated in plasma, urine, and kidney tissue of DN, which was enhanced in the DN progression. MiR-21 binds to target proteins and interacts with multiple signaling cascades, forming a complex network that promotes DN. Presently, more research are still warranted to obtain a better understanding of the clinicopathological features and the underlying mechanisms of miR-21, which could provide insights into the diagnostic, predictive, and therapeutic role of miR-21 in treating DN. The current evidence highlights an important area for future research focusing on the effective biomarkers for DN, which may facilitate early diagnosis and the judgment of prognosis for DN. This review advocates the pivotal role of miR-21 in the pathogenesis of DN, indicating that miR-21 could serve as a potential therapeutic target.

## Author Contributions

SL, WW, JL, and FT contributed to conceive and design the study. FT, GG, JP, XF, YZ, and ZC performed the systematic searching. SZ and WX extracted the data. SZ and WX wrote the manuscript. SL, WX, and SZ supervised the manuscript. All of the authors read and approved the final manuscript. All authors contributed to the article and approved the submitted version.

## Funding

This work was supported by the grants from the Zhejiang Medical and Health Science and Technology Program (No. 2022RC297, 2021PY085, and 2022KY1402); the Natural Science Foundation of Zhejiang Province (No. LQ22H040009); the Science and Technology Planning Project of Taizhou City, Zhejiang Province (No. 20ywb40, 20ywb45, and 1902ky43); the Project of Taizhou Central Hospital (Taizhou University Hospital) (NO. 2019KT015); High-level Hospital Construction Research Project of Maoming Peoples Hospital.

## Conflict of Interest

The authors declare that the research was conducted in the absence of any commercial or financial relationships that could be construed as a potential conflict of interest.

## Publisher’s Note

All claims expressed in this article are solely those of the authors and do not necessarily represent those of their affiliated organizations, or those of the publisher, the editors and the reviewers. Any product that may be evaluated in this article, or claim that may be made by its manufacturer, is not guaranteed or endorsed by the publisher.
